# Supplementary material for: The interactome of intact mitochondria by cross-linking mass spectrometry provides evidence for coexisting respiratory supercomplexes
Source: Mol Cell Proteomics. 2017 Dec 8;17(2):216–32. doi: 10.1074/mcp.RA117.000470 (PMC5795388; doi:10.1074/mcp.RA117.000470)
Supplement: Supplemental Data [file supp_RA117.000470_133922_0_supp_23978_fzffwf.zip › spectra_annotation/mito_DR_spectra_annotation/297-1-3-1-3-1.pdf]

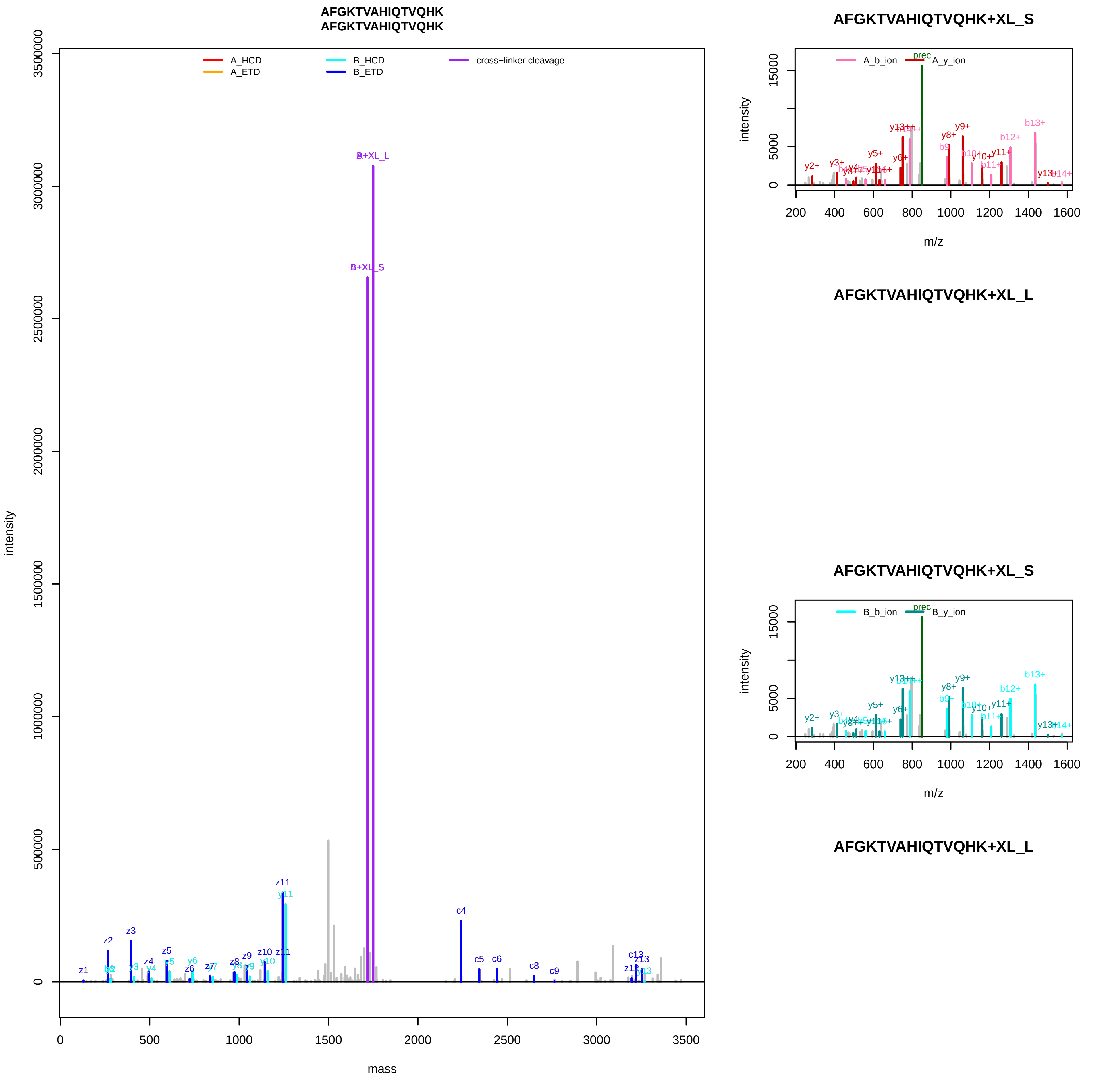

AFGKTVAHIQTVQHK
AFGKTVAHIQTVQHK
A_HCD
A_ETD
B_HCD
B_ETD
cross−linker cleavage
intensity
mass
B+XL_L
B+XL_S
z1
z2
z3
z4
z5
z6
z7
z8
z9
z10
z11
z12
z13
y5
y6
y8
y9
y10
y11
c4
c5
c6
c8
c9
c13
AFGKTVAHIQTVQHK+XL_S
A_b_ion
A_y_ion
prec
m/z
AFGKTVAHIQTVQHK+XL_L
AFGKTVAHIQTVQHK+XL_S
B_b_ion
B_y_ion
prec
m/z
AFGKTVAHIQTVQHK+XL_L
